# The World Health Organization African region external quality assessment scheme for anti-HIV serology

**DOI:** 10.4102/ajlm.v1i1.39

**Published:** 2012-11-07

**Authors:** Fatim Cham, Mahlatse Maleka, Martin Masango, Emma Goetsch, El H. Belabbes, Beverley Singh, Guy M. Gershy-Damet, Adrian Puren

**Affiliations:** 1 World Health Organization Regional Office for Africa, Brazzaville, Republic of Congo; 2 Centre for HIV and STI, National Institute for Communicable Diseases, South Africa; 3 Division of Virology and Communicable Disease, University of the Witwatersrand, South Africa

## Abstract

A regional external quality assessment scheme (REQAS) for anti-HIV serology aimed to objectively assess reliability and quality of HIV testing processes in the African region. This involved the distribution of proficiency testing (PT) panels to participating laboratories from 2002 to 2010. During the survey period, this included 16 distributions of PT panels to 49 laboratories in 30 countries, and the overall average score during the nine-year survey period was 98.9%, with a frequency of accurate detection, of anti-HIV-1 and/or anti-HIV-2 antibodies in the PT panels, ranging from 93% to 100%. Problems highlighted included lack of human resources and frequent stock outs of test kits, reagents and consumables for routine HIV testing. The design of the REQAS allowed appraisal of the reliability of anti-HIV serological testing methods utilised by laboratories for clinical assessment of patients and/or surveillance programmes. The REQAS was able to demonstrate that laboratories participating in the REQAS performed well and sustained their participation in the scheme. This bodes well for clinical diagnosis, surveillance and training activities at these reference laboratories.

## Introduction

Since the 2001 United Nations General Assembly 26th Special Session (UNGASS) declaration of commitment for access to treatment care and support services for people living with HIV and/or AIDS, there has been an unprecedented scaling up of integrated and comprehensive services for diseases of public health importance in the African region (www.UNAIDS.org)^1^. As laboratory and non-laboratory testing for HIV, TB and malaria is one of the main entry points of access to prevention and support services, accurate and reliable laboratory results are essential for diagnosis and monitoring of diseases of public health importance.^2^

In accordance with World Health Organization Regional Office for Africa (WHO AFRO) resolutions endorsed by member states to strengthen laboratory capacity in the African region, it was recommended that National Public Health Reference Laboratories (NPHRLs) develop and implement integrated Quality Management Systems (QMS) including participation in external quality assessment schemes (EQAS) for all diagnostic and monitoring tests. Additional recommendations included the implementation of integrated, cost-effective and sustainable national EQAS. Moreover, NPHRLs strengthening of their QMS is a gateway to identifying training needs and technical support in addition to attaining accreditation based on international standards.^3,4,5,6^

External quality assessment (EQA) is an objective means of assessing the integrity of the entire laboratory testing process, and aims to educate and improve performance in quality assurance (QA) issues.^7^ EQA includes, among others, on-site assessments, and blinded rechecking of previously tested specimens and/or proficiency testing.^8^ Proficiency testing (PT), an essential component of EQA, is an independent means of assessing the quality of the testing process whereby multiple well-characterised specimens are periodically sent to a group of laboratories for analysis and/or testing using routine laboratory procedures. Thus, PT programmes can assist laboratory services to identify factors contributing to errors, in addition to determining whether a laboratory can reliably perform a given test when compared to its peers.

In the African region, few public health laboratories are participating in EQAS for diagnostic and monitoring tests. These laboratories are for the most part limited to central level laboratories, mainly because schemes coordinated by international or regional providers are not able to provide sufficient advice on remedial actions when necessary. Moreover, these schemes are relatively expensive for national governments to extend participation to laboratories in the tiered laboratory network. Hence, in response to requests from member states, WHO AFRO established the regional external quality assessment schemes (REQAS) for anti-HIV serology in collaboration with the National Institute of Communicable Disease (NICD) in South Africa and the National Reference Laboratory (NRL) in Senegal to support the Anglophone and Francophone countries, respectively. The REQAS, established in March 2002, aim to assess the quality of anti-HIV-1 and HIV-2 serological testing for interested laboratories. Additionally, the REQAS allow comparison of testing facilities, in addition to evaluating the quality of serological testing using enzyme immunoassays (EIA) and HIV rapid tests. Participation in the REQAS is voluntary and at no cost to laboratories, thus allowing participation of laboratories in at least one of the REQAS components. The scope of this paper is limited to the REQAS for anti-HIV serology coordinated by NICD and aims to present results of surveys conducted from 2002 to 2010 and challenges encountered. The present article describes results of a REQAS for anti-HIV serology established for public health laboratories in the African region and discusses the implications for efforts aimed at assuring the quality of HIV testing and strengthening public health laboratories towards accreditations in the African region.

## Ethical considerations

These laboratories applied to participate in the surveys.

## Methods

### Characterisation of bulk volume specimens

Bulk volume blood obtained as plasma from the South African National Blood Services was converted to serum by recalcification and heat-inactivated at 56 °C for 60 minutes.^9^ Serum samples were characterised by testing on at least three different 3rd generation anti-HIV-1/2 enzyme immunoassays (EIAs), three different anti-HIV-1/2 rapid tests and by Western Blotting.

From 2002 to 2010, a total of 17 PT panel batches (001–016), each comprised of ten serum specimens, were dispatched to laboratories. The PT panel batches 001–013, 015–016’ were comprised mainly of HIV-1 sero-positive and sero-negative samples. For PT panel batch 014, two batches (014–1 and 014–2) were dispatched: PT panel batch 014–1 was comprised of four HIV-1 and six sera-negative samples and PT panel batch 014–2 included two HIV-2, four HIV-1 and four HIV sero-negative samples. PT panel batch 014–2 was dispatched to 14 laboratories that responded to a survey confirming capacity to sero-type HIV-2. HIV-2 sero-positive samples were characterised by ELISA and confirmed using the Multispot Rapid Test kit (Bio-Rad laboratories, USA) and Western Blot using NEW LAV BLOT II (Bio-Rad laboratories, USA). All assays used in the characterisation of the PT panels were performed according to manufacturer’s instructions.

Furthermore, the *env* subtype of 38 HIV sero-positive samples included for PT panel batches 001 to 008 was determined by Heteroduplex Mobility Assay (HMA). Of these, 36 were *env* subtype C whilst 2 were *env* subtype B. Due to logistic and cost reasons, *env* sub-typing of panels was discontinued for PT panel batches 009 to 016. However, it is likely that panels sourced from the South African blood bank were either subtype B or C based on results of surveys of HIV-1 subtype conducted in South Africa.^10^

Characterised serum panels were aliquoted in 100µl volumes, labelled and stored at −80 °C until panels were ready for shipment to laboratories. The PT panels containing ten serum samples of known anti-HIV status together with instructions and report forms were distributed to participating laboratories twice a year, in March and October.

In 2002 and 2004, the PT panels were dispatched once to 17 and 34 laboratories, respectively. However, the panels were sent out twice yearly in 2003 and from 2005 to 2010. For each shipment, ten serum specimens in United Nations (UN) approved packages including instruction notes and reporting forms were transported to laboratories by an IATA-certified company.^11^ To reduce shipping costs to countries with more than one participating laboratory, PT panels were shipped to the respective central level laboratories for onward distribution to other regional or peripheral laboratories. Furthermore, the central level laboratories were also responsible for collating results of the PT testing and reporting to the REQAS coordinators. Laboratories were instructed to return results by either fax or e-mail to the REQAS coordinators within 30 days of receipt of the panels. Additionally, upon receipt of data from laboratories, the REQAS coordinator forwarded the expected results and reports of individual laboratory performance scores on the PT panels. In addition, a distribution report comprising comparative data from all laboratories, complete results of the panel characterisation, test readings as well as methodologies used for testing each panel preparation was generated and forwarded to laboratories.

### Utilisation of internal quality control

Laboratories were requested to complete a questionnaire on their use of internal quality control (IQC) materials. The purpose of the questionnaire was to determine whether IQC was routinely performed and if it was, whether laboratories were using test kit controls supplied in the test kit or ’in-house’ produced controls.

### Analysis of data

PT panels were scored based on assigned HIV serology positive or negative status of each PT sample as characterised by the EQAS provider. Results that were discordant from the expected result were assigned 0 points whilst concordant results were assigned 2 points with a total possible score of 20 points for 10 samples in each PT batch. In addition, a combined score was calculated according to participant and a combined score was obtained for the group as well as the coefficient of variation of the score over time.

## Results

### PT panel testing methodologies

Responses relating to testing methodologies received from laboratories indicated a wide range of platforms used for screening and confirmation of anti-HIV antibodies. The WHO recommends three testing strategies (I, II and III) which aim to increase accuracy viz., prevalence or diagnostic testing whilst reducing costs for determining HIV sero-status^12^. Laboratories select the most appropriate strategy, based on prevalence and purposes of testing. Only eight laboratories out of 49 participants confirmed that they are currently using WHO Strategy II and III, for HIV prevalence testing with serial or parallel testing algorithms for diagnostic purposes. Three laboratories out of 49 participants reported using WHO Strategy II with serial testing algorithm, whereas 21 laboratories reported using the serial testing algorithm only. Five laboratories reported using WHO testing strategy III, which differs from strategy II as it employs a third assay platform based on different antigen preparations and/or different test principles from assay platforms used in screening tests^12^.

**FIGURE 1:**
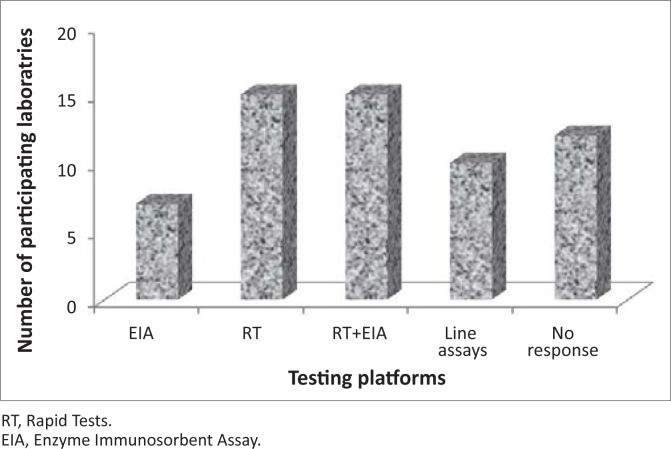
Platforms used by participating laboratories to test the proficiency testing panels 2002-2010.

Of the 49 laboratories, 15 performed rapid HIV testing only whilst seven performed ELISA testing only. Fifteen laboratories performed both ELISA and rapid HIV testing. Ten of 49 laboratories reportedly used line probe assays. Of these, four laboratories used Western Blot assays, four used Inno-lia assay and two used the HIV Blot 2.2. Twelve laboratories did not report on what type of testing was used (Figure 1).

### Participation and response rate of laboratories

**FIGURE 2:**
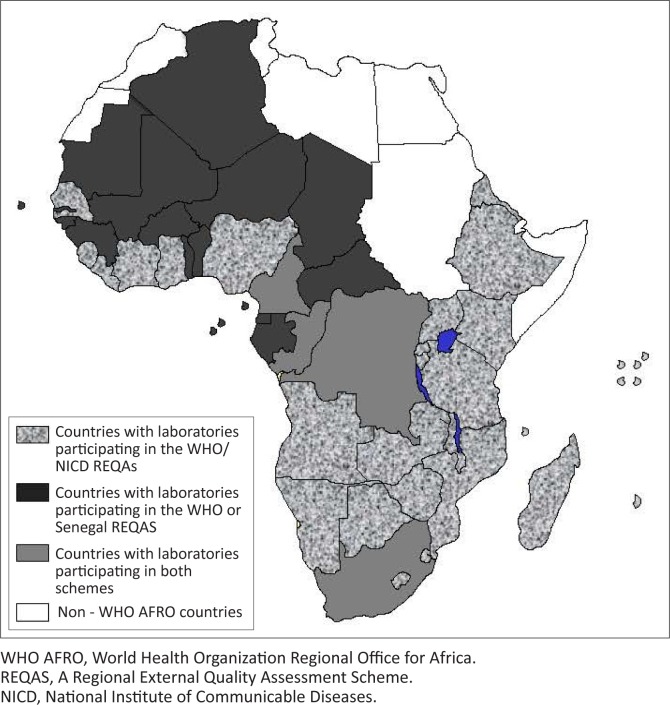
Countries currently participating in the WHO/REQAS for anti-HIV serology.

The REQAS has to date registered 49 laboratories in 30 African countries (Figure 2). Thirteen countries, including Botswana, Comoros, Eritrea, Ivory Coast, Kenya, Malawi, Mozambique, Rwanda, Senegal, the United Republic of Tanzania, Uganda, Zambia and Zimbabwe, have more than one laboratory participating in the REQAS. From 2005 to 2010, 14 laboratories were newly enrolled in the scheme, including laboratories in Angola, Burundi, Ivory Coast, Kenya, Liberia, Malawi, Rwanda, Sierra Leone, Uganda, the United Republic of Tanzania and Zambia. In 2010, five regional laboratories in Tanzania, currently preparing for accreditation, enrolled in the REQAS. Laboratories in Cameroon, the Democratic Republic of Congo (DRC), the Republic of Congo and South Africa were subscribing to the REQAS coordinated by both NICD and the NRL in Senegal.

**FIGURE 3:**
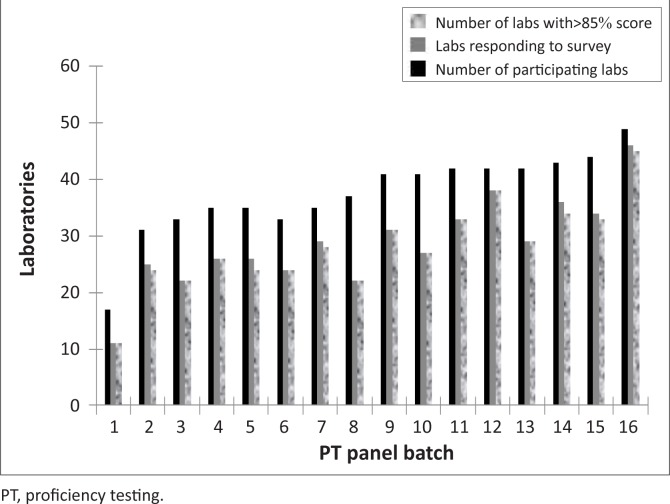
Summary of EQA results for the survey period during 2002–2010.

At the start of the surveys, 17 laboratories enrolled in the REQAS; however the number of participating laboratories increased more than two-fold with 49 laboratories participating at the end of 2010. The response rate by participating laboratories was also observed to increase from 65% for PT panel batch 001 in 2002 to 95% for PT panel batch 016 in 2010. However, the response rate declined to 60% for PT panel batch 008 (Figure 3). Although, the need for routine testing of PT panels was emphasised by the REQAS coordinators, in several facilities PT panels were not processed according to the routine testing algorithm or results of the PT panels were not submitted within the 30-day deadline stipulated by the REQAS coordinators. For most laboratories, failure to use the national testing algorithm and/or to return results on time was mainly due to lack of sufficient financial resources, reagents and/or non-functional equipment to complete testing at the time of the external assessment. Hence, in some cases laboratories tested PT samples using only one test kit, as opposed to the routine testing algorithm of the laboratory, whilst others reported using expired test kits. Additional problems encountered related to improper handling and processing of PT panels as well as inadequate human resources, which invariably affected the post-analytical stages of data reporting and analysis.

### PT panel testing results

Within the 30-day deadline, participating laboratories returned results of the PT panels to the coordinators of the REQAS via email, fax or regular postage. A zero and two score was assigned for discordant and concordant results, respectively. Hence a maximum score of 20 was attainable if participating laboratories results were 100% concordant with the expected results per distribution. A score ≥ 85% (≥ 17/20) was set as the cut-off for acceptable performance and a root cause analysis (RCA) for unacceptable results was conducted for laboratories scoring ≤ 16/20. An unassigned score for a particular distribution indicates non-participation, late responses or non-return of results.

**FIGURE 4:**
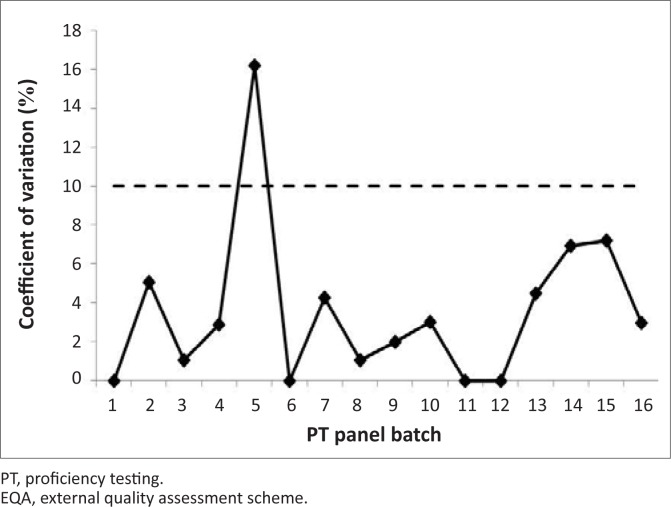
Coefficient of variation (%) of EQA results during the survey period (2002–2010).

During the survey period, 42 laboratories attained the passing score (≥ 85%) with only seven laboratories receiving unacceptable scores (Figure 3). The overall average score during the survey period was 99% (19.8/20) with a coefficient of variation (CV%) of < 10% for all distributions, with the exception of PT panel batch 005. The CV% for PT panel batch 005 was 16.2% (Figure 4).

Inter-laboratory variations were mainly due to reporting of discordant results that caused an overall decrease in the average score for PT panel batches 005, 010, 013, and 014, with PT panel batch 013 having the lowest average score of 19 out of 20. Moreover, the high CV% for PT panel batch 005 was as a result of 20 discordant results from nine laboratories. Of these discordant results, 30% were due to discordant results reported for a panel sample characterised as HIV sero-negative.

For PT panel batch 013, a panel sample characterised as HIV-1 sero-positive was reported as sero-negative by 12 out of 29 laboratories. To establish the root cause, the sample was included in PT panel batch 014–1 and 014–2. It was noted that 13 out of 36 laboratories still reported the sample as sero-negative. Results of the RCA indicated that laboratories failing to correctly report the sero-status of this sample for PT panel batch 013 and 014 used a fourth-generation ELISA kit as part of their testing algorithm. Additionally, some laboratories also reported faint bands with rapid tests and falsely interpreted the sample as HIV-1 sero-negative.

Thirteen out of 14 laboratories that received PT panel batch 014-2 correctly identified the two HIV-2 specimens. One laboratory failed to return results to the REQAS coordinators.

For PT panel batch 010, some laboratories reported receiving leaked or empty samples upon arrival of the PT panel shipment. However, these samples were excluded from the overall scoring. Hereafter, the REQAS coordinators discontinued the use of these tubes for subsequent distributions.

### Internal quality control

Results based on responses to the questionnaires relating to IQC indicated that 73% of laboratories routinely used IQC materials. Of these, 92% used ‘in-house’ prepared controls, whereas 8% reported using IQC materials supplied with the test kits. Furthermore, of the laboratories that responded using ‘in-house’ IQC material, 58% indicated that a single serum or plasma specimen was utilised as IQC material whilst 39% of the responding laboratories used multiple sera and/or plasma and 1 lab used ‘other’ (positive control is diluted with the negative control to make a weakly positive). Furthermore, 45% of laboratories indicated that IQC materials were included in each EIA plate run, 34% of the laboratories reported using IQC with each new batch of test kits, 16% of laboratories reported that IQC materials were included daily, and 5% of laboratories reported including IQC on weekly basis.

## Discussion

The comparative performance data generated by the REQAS illustrates the added value of quality assured laboratory services to strengthening health systems. In general, the REQAS demonstrated that public health laboratories are able to accurately determine the HIV sero-status of clinically derived specimens, within the defined parameters of the scheme. The format of HIV serology testing methods varied over the survey period; microplate-based EIAs were mostly used at the start of the survey but were increasingly replaced with simple and rapid testing methods. The switch may have been driven by the numbers of specimens tested daily and/or budgets of participating laboratories.

Retrospective evaluation over the nine-year survey period indicated a 98.8% overall concordance between reported and expected results from participating laboratories, with the highest percent of overall errors found in EIAs. Noticeably, during the survey period, fourth-generation (i.e. combined detection of HIV antigen and antibody) EIA test kits were observed as giving the most discordant results. Interestingly, most laboratories using the HIV Vironostika Uni-form Ag/Ab (BioMérieux, France) switched to using either the Murex HIV 1.2.0 (DiaSorin, Italy) or the Vironostika HIV Uniform II PLUS O (BioMérieux, France). With the exception of challenges with rapid tests kits used by a few laboratories reporting discordant results for PT panel batches 013 and 014, minimal errors appeared with other testing platforms.

The reason for discordant and/or equivocal results with the fourth-generation EIAs is not clear and is beyond the scope of this paper. However, PT panels were characterised using third-generation assays, Genscreen HIV 1/2 (Bio-Rad, Germany), Vironostika Uniform II Plus O (BioMérieux, France) and Murex HIV 1.2.O (DiaSorin, Italy), hence it is important to determine the process of achieving cut-offs for testing using fourth-generation assays to understand the difference in performance to third-generation assays. Other sources of false reporting may be due to inappropriate PT panel storage conditions upon receipt, test kit storage and handling, use of expired test kits as well as failure to follow the routine testing algorithm.

Testing algorithms are defined as the combination and sequence of specific test kits used in a given testing strategy; it also describes the sequence of tests to be performed. The key to achieving the final result is to always follow the sequence of the tests in the algorithm. From results of the nine-year survey, it was apparent that most laboratories failed to adhere to their national algorithms. Some laboratories relied on one test for determining HIV-1 sero-status, whilst others were performing additional testing on PT specimens found to be non-reactive on the first screening test, which often led to inconclusive overall results. Furthermore, laboratories indicated that confirmatory tests were routinely performed on site for all reactive specimens. Hence, it was noted that 95% of laboratories performed confirmatory testing on reactive samples that were part of the PT panel whilst 5% reported performing screening tests on the PT panels. Additionally, some laboratories failed to realise the importance of ongoing participation in an EQAS, proper maintenance of records and application of corrective actions to improve testing services.

To address the issues of non-response by participating laboratories which were classified as either panel received but not processed, panel received but problems with testing (either routine or panel-specific) or panel processed but results not submitted or submitted late, the coordinators of the REQAS continued to encourage laboratories to report problems encountered with testing of the PT panels, in case assistance is required in resolving a specific laboratory problem. Furthermore, the REQAS coordinators established the RCA concept for laboratories. RCA aims to determine the reasons(s) for an inadequate or poor performance as well as non-responses to allow the REQAS coordinators to provide the required technical support to solve problems that exist in the laboratories. On the other hand, the RCA allowed laboratories to assess whether the PT panels were received in a satisfactory condition or whether the correct samples were tested using the appropriate method as stipulated in their site-specific standard operating procedures.

The REQAS is at no cost to participating laboratories, as the scheme is funded mainly by WHO AFRO through donor support. With increasing participation of laboratories, the REQAS using liquid serum panels may not be sustainable, especially with high cost of courier transport particularly with the requirements for cold chain transport. To alleviate these challenges, the REQAS coordinators plan to pilot dried tube specimen (DTS) technology in 2011. DTS technology is a practical and cost effective methodology for assuring the quality of serological testing in resource limited settings ^13^. Another limitation of the REQAS was the distribution of mostly HIV-1 *env* subtype B and C panels to laboratories that routinely test non-subtype B or C samples. Henceforth, the coordinators of the scheme have considered inclusion of non-subtype C HIV-1 and HIV-2 sera as part of the PT panel batches.

In summary, analysis of results of the REQAS presented in this report provides evidence that public health laboratories in the African region are capable of accurately determining HIV sero-status albeit with challenges related to human and financial resources. Moreover, the high quality of results is evidence that the majority of African laboratories have the capacity to provide quality-assured data for clinical assessment of patients and/or surveillance programmes.
